# Bleeding in Affections of the Brain

**Published:** 1840-01

**Authors:** 


*Bleeding in Affections of the Brain.*— Is not depletion by bleeding a practice still too general and indiscriminate in affections of the brain, and especially in the different forms of paralysis? I believe that the soundest medical experience will warrant this opinion. The vague conception that all these disorders depend upon some inflammation or pressure, which it is needful to remove, too much pervades and directs the prctice in them: and, if the seizure be one of sudden kind, this method of treatment is often pursued with an urgent and dangerous activity. Little heed is taken of the many cases where the symptoms depend upon irritation alone--or on loss of nervous power—or on deficient circulation of the blood within the brain—or on altered qualities of this blood—or, it may be, on morbid changes in the nervous substance itself. Theory might suggest that, in some of these various cases, the loss of blood leads to mischief.—Experience undoubtedly proves it; and there is cause to believe that this mischief, though abated of late years, is still neither infrequent nor small in amount.

It is certain indeed that there is a state of brain, best perhaps represented to us in its general effects of diminished nervous power, which tends to produce sometimes spasmodic seizures, sometimes delirious or maniacal affections, sometimes palsy of different parts of the body—these effects being in no wise obviated by depletion, but rather increased by all such means; while they are relieved by remedies which tend to excite the energy of the sensorium, and to augment the general power.—*Ib.*

